# FGF, Mechanism of Action, Role in Parkinson’s Disease, and Therapeutics

**DOI:** 10.3389/fphar.2021.675725

**Published:** 2021-06-21

**Authors:** Yiqiu Liu, Junyu Deng, Ye Liu, Wei Li, Xuqiang Nie

**Affiliations:** ^1^ College of Pharmacy, Zunyi Medical University, Zunyi, China; ^2^ Joint International Research Laboratory of Ethnomedicine of Chinese Ministry of Education, College of Pharmacy, Zunyi Medical University, Zunyi, China; ^3^ Key Lab of the Basic Pharmacology of the Ministry of Education, College of Pharmacy, Zunyi Medical University, Zunyi, China

**Keywords:** fibroblast growth factor, Parkinson’s disease, signaling pathways, α-synuclein, dopaminergic neurons

## Abstract

Parkinson’s disease (PD) is a neurodegenerative disease associated with severe disability and adverse effects on life quality. In PD, motor dysfunction can occur, such as quiescence, muscle stiffness, and postural instability. PD is also associated with autonomic nervous dysfunction, sleep disorders, psychiatric symptoms, and other non-motor symptoms. Degeneration of dopaminergic neurons in the substantia nigra compact (SNPC), Lewy body, and neuroinflammation are the main pathological features of PD. The death or dysfunction of dopaminergic neurons in the dense part of the substantia nigra leads to dopamine deficiency in the basal ganglia and motor dysfunction. The formation of the Lewy body is associated with the misfolding of α-synuclein, which becomes insoluble and abnormally aggregated. Astrocytes and microglia mainly cause neuroinflammation, and the activation of a variety of pro-inflammatory transcription factors and regulatory proteins leads to the degeneration of dopaminergic neurons. At present, PD is mainly treated with drugs that increase dopamine concentration or directly stimulate dopamine receptors. Fibroblast growth factor (FGF) is a family of cellular signaling proteins strongly associated with neurodegenerative diseases such as PD. FGF and its receptor (FGFR) play an essential role in the development and maintenance of the nervous system as well as in neuroinflammation and have been shown to improve the survival rate of dopaminergic neurons. This paper summarized the mechanism of FGF and its receptors in the pathological process of PD and related signaling pathways, involving the development and protection of dopaminergic neurons in SNPC, α-synuclein aggregation, mitochondrial dysfunction, and neuroinflammation. It provides a reference for developing drugs to slow down or prevent the potential of PD.

## Introduction

Parkinson’s disease (PD) is considered the most common neurodegenerative disease after Alzheimer’s disease (Feigin et al., 2017). In Asia, Africa, Europe, North America, South America, and Arab countries, the crude prevalence rates of PD for all age groups are 15–119 per 100,000, 10–43 per 100,000, 66–1,500 per 100,000, 111–329 per 100,000, 31–470 per 100,000, and 27–43 per 100,000, respectively (Kalia and Lang, 2015). Age is the most significant risk factor for the development of PD. The prevalence and incidence of PD almost increase exponentially with age, reaching its peak after 80 years old (Driver et al., 2009; Pringsheim et al., 2014). With the aging of the world’s population, PD will cause an increasing social and economic burden on society. In 2016, an estimated 6.1 million people worldwide were diagnosed with PD, which is 2.4 times that of 1990 (Dorsey et al., 2018). It is estimated that by 2040, the global prevalence of PD will double (Dorsey and Bloem, 2018). The overall prevalence rate and annual incidence rate of PD in China are 190/100,000 and 362/100,000, respectively, which are lower than those in developed countries but higher than those in some developed countries (Ma et al., 2014).

PD usually has two main features: 1) the death of dopaminergic (DA) neurons in the pars compacta of substantia nigra, and 2) misfolded α-synuclein (α-syn) accumulates in neuronal cell bodies or dendrites and axons to form Lewy bodies (LBs) or Lewy neurites (LNs) (Goedert et al., 2013). α-synuclein aggregates are harmful to dopaminergic neurons in the substantia nigra. Their formation may trigger the transfer of toxic α-synuclein from affected cells to other neighboring cells, resulting in a cascade of LBs formation, leading to cell death (Emamzadeh and Surguchov, 2018). The diffusion of pathological α-synuclein to adjacent cells leads to the progressive loss of dopaminergic neurons in SN, accompanied by a decrease in dopamine levels, and eventually leads to dyskinesia (Luk et al., 2012).

PD is usually divided into two subtypes: tremor-predominant PD and non-tremor-predominant PD (including severe motor syndrome and postural instability and gait difficulties), and tremor-dominant PD progresses more slowly and has milder dysfunction than non-tremor-dominant PD (Thenganatt and Jankovic, 2014). PD is also related to many non-motor symptoms, including pain, loss of smell, psychotic features, sleep disorder, and autonomic nerve dysfunction, which usually occur before motor symptoms and sometimes last for many years (Schapira et al., 2017). At present, PD mainly uses drugs that increase dopamine concentration or directly stimulate dopamine receptors for symptomatic treatment. Although drug treatment can effectively control many symptoms, there is still a significant risk of adverse events in long-term treatment, such as levodopa-induced dyskinesia (Turcano et al., 2018) and dopamine agonist-induced impulse control disorder (ICDs) (Garcia-Ruiz et al., 2014).

Withdrawal symptoms are easy to appear after drug withdrawal (Pondal et al., 2013). In addition, these drugs will not change the course of the disease, and with the development of PD, its symptomatic benefits will decrease, so patients need to increase the frequency and dosage of drugs, which increases the risk of adverse events (Armstrong and Okun, 2020). The lack of adequate disease remission treatment may reflect the multifactorial nature of the underlying pathogenesis of PD. Including oxidative stress and mitochondrial dysfunction, protein misfolding and aggregation, neuroinflammation and excitotoxicity, etc. (AlDakheel et al., 2014). Therefore, the development of drugs that can provide neuroprotection or repair in PD not only has a significant advantage over existing treatments but may also help to prolong its validity.

Fibroblast growth factors (FGFs) is a secreted protein family with a wide range of signal molecular functions in angiogenesis, embryonic development, cell proliferation, and wound healing (Beenken and Mohammadi, 2009). In recent years, with the in-depth study of FGF family, the role and mechanism of FGF in brain-related diseases have attracted much attention. Many studies have proved that FGF and its receptors play a key role in neuroprotection and neurogenesis of PD, including proliferation and differentiation of stem cells during development and in the adult brain. In PD model, FGF can provide effective protection against dopaminergic neuron loss, promote the development and survival of nervous system, relieve neurological symptoms and exert neurotrophic activity on DA neurons *in vivo* and *in vitro*. These findings indicate the importance of FGF in the differentiation and survival of dopamine neurons, and the etiology and treatment of PD (Ye et al., 1998; Tanaka et al., 2001; Timmer et al., 2007; Mäkelä et al., 2014). In the following, this review will provide an overview of this growth factor family, summarize its significance in the pathophysiology of PD, and discuss possible opportunities for targets to obtain new treatment strategies.

## The Fibroblast Growth Factors

In 1973, Armelin purified a cell growth factor from pituitary extracts, which was named as fibroblast growth factor because of its ability to promote the proliferation of fibroblasts, and was determined to have an isoelectric point of 9.6, so it was called basic fibroblast growth factor (bFGF or FGF-2) (Armelin, 1973). Subsequently, the substance causing proliferation of fibroblasts in bovine brain extract was found in bovine brain extracts, which was identified as having different FGF-like activities, and was called acidic fibroblast growth factor (aFGF or FGF-1) because its isoelectric point was 5.6 (Gospodarowicz, 1974). Genome sequencing of humans and mouse showed that there were 23 members of the mammalian FGF family. FGF 11–14 are not always included in the FGF family. However, they have a high amino acid sequence identity with the FGF family and bind heparin with high affinity. They have no ability to bind fibroblast growth factor receptor (FGFR) and activate fibroblast growth factor receptor, and they are called FGF homologous factors (Olsen et al., 2003). FGF-15 is a mouse ortholog of human FGF-19 (Beenken and Mohammadi, 2009).

**FIGURE 1:**
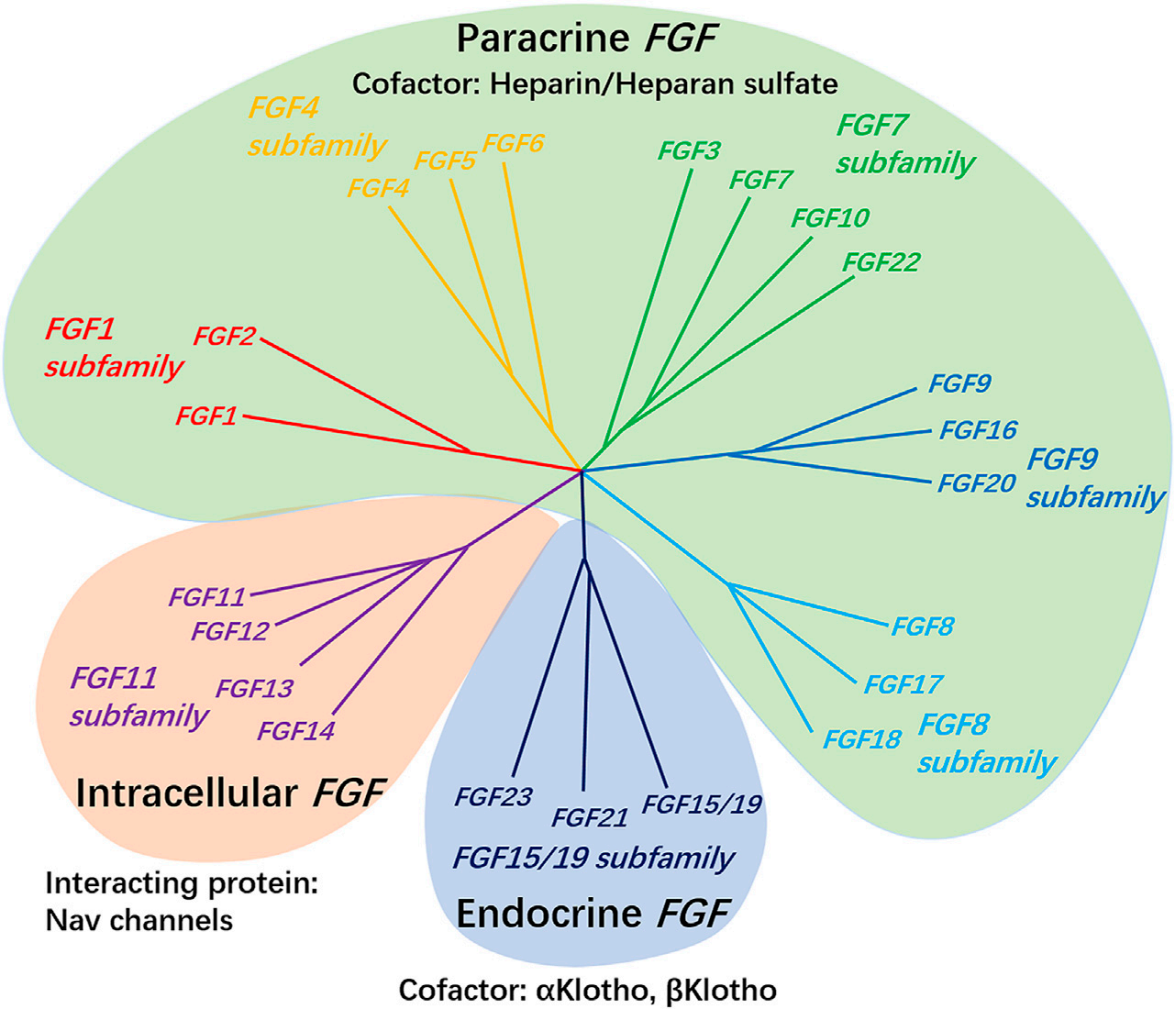
The FGF 1, FGF 4, FGF 7, FGF 8, and FGF 9 subfamily genes encode secreted paracrine FGFs, which bind to and activate FGFRs with heparin/HS as a cofactor. The FGF 15/19 subfamily members encode endocrine FGFs, which combine and activate FGFRs with the Klotho family protein as a cofactor. The FGF 11 subfamily genes encode intracellular FGFs, which are non-signaling proteins serving as cofactors for voltage-gated sodium channels and other molecules.

According to biochemical function, evolutionary relationships and sequence homology, FGF can be divided into seven subfamilies (Figure 1). It is composed of secreted fibroblast growth factor (including paracrine FGF 1–10, FGF 15–18, FGF-20, FGF-22, endocrine FGF 19/21/23) and intracellular fibroblast growth factor (FGF 11–14). The former sends signals to receptor tyrosine kinases, while the latter does not, and has no clear interaction with the signal transduction FGFRs, which is mainly acts as cofactors for voltage-gated sodium channels and other molecules (Ornitz and Itoh, 2001; Goldfarb, 2005; Itoh and Ornitz, 2008; Ornitz and Itoh, 2015). The special function of intracellular FGF may help to regulate the subcellular localization of in axon initiation segment during development, as well as the ion gating characteristics of other excitable cell channels such as mature neurons and cardiomyocytes (Goldfarb et al., 2007; Laezza et al., 2007; Wang et al., 2011; Xiao et al., 2013).

FGF subfamilies usually have similar expression patterns, although each FGF seems to have its own unique expression sites. Some secreted fibroblast growth factors are only expressed during embryonic development (such as FGF 3, 4, 8, 15, 17, and 19), and they act as necessary regulatory factors at the earliest stages of embryonic development and organ formation, while others are expressed in embryonic and adult tissues (such as FGF 1, 2, 5–7, 9–14, 16, 18, and 20–23), where they regulate growth and function as factors in tissue maintenance, repair, and regeneration, and endocrine FGFs have a key role in the regulation of postnatal phosphate, bile acid, carbohydrate, and lipid metabolism (Ornitz and Itoh, 2001; Ornitz and Marie, 2015). Members of secretory FGF subfamily can also be further characterized according to the mechanism of their release from cells. FGF 3–8, 10, 15, 17, 18, 21, 22, and 23 are secretory proteins with cleavable amino terminal signal peptide. FGF 9, 16, and 20 are also secreted proteins, but contain a non-cleavable dimeric secretory signal sequence, and it has been shown that hydrophobic sequences in their structure are critical for their secretion and can be transported to and from the endoplasmic reticulum as non-cleavable signals (Itoh and Ornitz, 2011). In contrast, FGF-1 subfamily has no recognizable signal sequence, so it does not secrete FGF-1 and FGF-2, but it can still be found in extracellular position, which is mainly exported from the cell through the cell membrane through direct translocation (Prudovsky et al., 2013). In addition, FGF-1 and FGF-2 were also found in some cell nuclei. Potential functions of nuclear FGF-1 include regulating cell cycle, cell differentiation, survival and apoptosis (Pirou et al., 2017).

**FIGURE 2:**
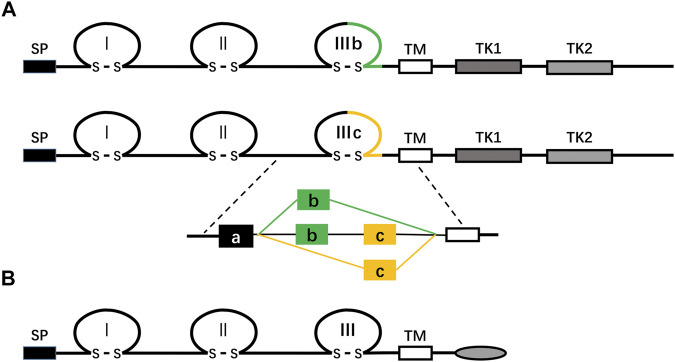
**(A)** shows a schematic diagram of the protein structure of FGFR. FGFR is a receptor tyrosine kinase composed of about 800 amino acids, with multiple domains, including three extracellular immunoglobulin-like domains (I, II, and III), a transmembrane domain (TM), and two intracellular tyrosine kinase domains (TK1 and TK2). SP represents a cleavable secretory signal sequence. The FGFR gene family consists of four members, FGFR 1–4. Among them, FGFR 1–3 produces two major splicing variants of the immunoglobulin-like domain III, called IIIb and IIIc, which are essential determinants of ligand binding specificity. **(B)** The schematic representation of the FGFRL1/FGFR5 protein structure is shown. FGFR5, similar to FGFRs in structure, is a membrane protein composed of about 500 amino acids, with three extracellular immunoglobulin-like domains (I, II, and III), a transmembrane domain (TM), and a short cytoplasmic tail without tyrosine kinase domain. SP represents a cleavable secretory signal sequence.

The FGFR family consists of four highly conserved transmembrane tyrosine kinase receptors (FGFR 1–4) and one receptor (FGFR5, also known as FGFRL1) that can bind to FGF ligands but lacks the intracellular protein tyrosine kinase domain (Figure 2; Balestrino and Schapira, 2020; Ornitz and Itoh, 2015; Trueb, 2011). FGFR is composed of three critical domains: extracellular ligand-binding domain, the single transmembrane domain, and intracellular protein tyrosine kinase domain (Tiong et al., 2013). The extracellular ligand-binding region contains three immunoglobulin (Ig)-like domains: Ig-I, Ig-II, and Ig-III (also known as D1, D2, and D3) (Tiong et al., 2013). In FGFR1-3, through alternative splicing of the IgIII domain (D3), each receptor’s IIIb and IIIc subtypes are generated, which have different ligand binding and cell and tissue expression specificities (Gong, 2014; Table 1). For example, FGFRb splicing variants occur mainly in epithelial tissues and bind to FGFs expressed in mesenchymal tissues, while FGFRc splicing variants exist in mesenchymal tissues and bind to FGF ligands expressed in both epithelial and mesenchymal cells (Gong, 2014). FGFR5 is similar to other FGFRs in structure, but it lacks the domain of intracellular protein tyrosine kinase, which is replaced by the intracellular tail of short cells rich in histidine motifs. Therefore, FGFR5 cannot transmit signals through trans autophosphorylation, nor can it play a role like other FGFRs (Trueb, 2011).

**TABLE 1:** Receptor specificity of FGFs.

FGF subfamily	FGF	Cofactor	FGF receptor activity
FGF1 subfamily	FGF1	Heparin or heparan sulfate	All FGFRs
	FGF2		FGFR1c,3c > 2c,1b,4
FGF4 subfamily	FGF4		FGFR1c,2c > 3c,4
	FGF5		FGFR1c,2c > 3c,4
	FGF6		FGFR1c,2c > 3c,4
FGF7 subfamily	FGF3		FGFR2b > 1b
	FGF7		FGFR2b > 1b
	FGF10		FGFR2b > 1b
	FGF22		FGFR2b > 1b
FGF8 subfamily	FGF8		FGFR3c > 4>2c > 1c >> 3b
	FGF17		FGFR3c > 4>2c > 1c >> 3b
	FGF18		FGFR3c > 4>2c > 1c >> 3b
FGF9 subfamily	FGF9		FGFR3c > 2c > 1c, 3b >> 4
	FGF16		FGFR3c > 2c > 1c,3b>>4
	FGF20		FGFR3c > 2c > 1c,3b>>4
FGF19 subfamily	FGF19	β-Klotho	FGFR1c,2c,3c,4
	FGF21		FGFR1c,3c
	FGF23	α-Klotho	FGFR1c,2c,3c,4
FGF11 subfamily	FGF11	Is not combined with FGFRs	
	FGF12		
	FGF13		
	FGF14		

The interaction of the FGF ligand and its signal receptors is regulated by proteins or proteoglycan cofactor and extracellular binding proteins (Kuro-o, 2008; Shimokawa et al., 2011). Paracrine FGF combined with FGFR to form FGF-FGFR-HS ternary complex in a heparin/heparan sulfate proteoglycan (HSPGs) dependent manner, which increased the affinity of FGF to FGFR, stabilized the formation of a dimer and enhanced the activation of the receptor (Schlessinger et al., 2000). Compared with paracrine FGFs, endocrine FGFs have a low affinity for heparin/heparan sulfate and can freely spread from the cells that secrete them, enter the blood circulation, and reach the target cells in distant organs (Goetz et al., 2007). Endocrine FGF depends on Klotho proteins (α-Klotho and β-Klotho) as the primary tissue-selective cofactor to promote the high-affinity binding of FGF ligand with its homologous FGFR, and then activate FGFRs (Kurosu and Kuro, 2009; Goetz et al., 2012).

**FIGURE 3:**
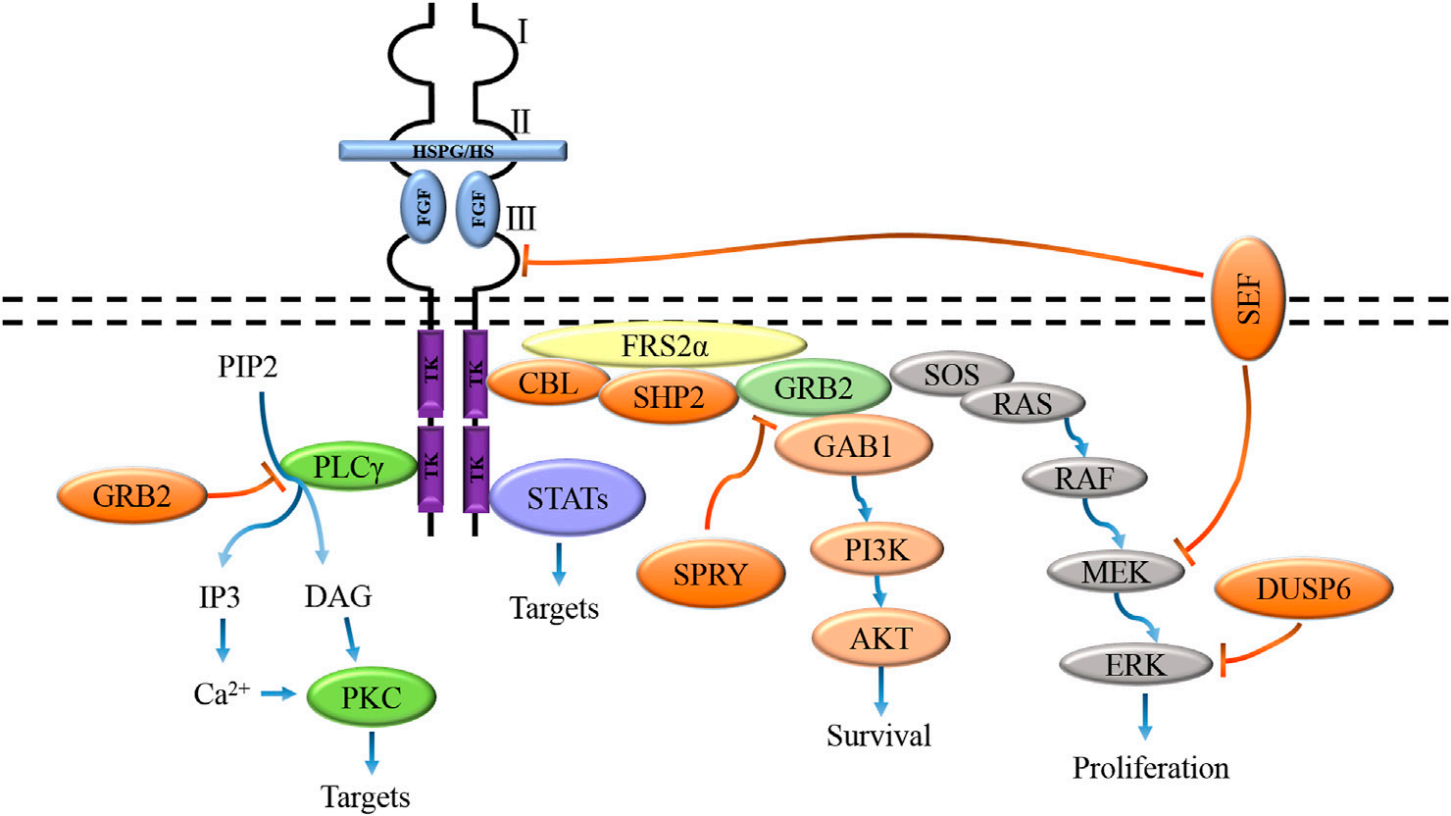
FGFs activate signaling pathways coupled to the cell by interacting with specific FGFRs and HS/HSPGs to activate receptors, including the RAS-MAPK, PI3K-AKT, PLC-γ, and STAT pathways. These pathways are negatively regulated by GRB2, DUSPs, SPRY, SEF, and CBL activities.

The biological activity of FGF is mediated by combining FGFR to initiate intracellular signal transduction. The binding of FGF and receptor induces dimerization of FGFR, which makes the protein tyrosine kinase domains close to each other and locate correctly, thus activating the kinase through trans autophosphorylation. Activated FGFR kinase activates its intracellular substrate through phosphorylation and initiates different but possibly interactive signal pathways, resulting in different cell reactions and functions (Furdui et al., 2006; Lemmon and Schlessinger, 2010). Activation of FGFR tyrosine kinase domain results in phosphorylation of junction proteins of four major intracellular signaling pathways, including RAS-MAPK, PI3K-AKT, PLC-γ, and signal transducer and transcriptional activator (STAT), in which the activation of RAS-MAPK and PI3K-AKT pathways is initiated by phosphorylation of fibroblast growth factor receptor substrate 2α (FRS2α) (Figure 3) (Ornitz and Itoh, 2015). Tyrosine-phosphorylated FRS2α functions as a coordinated assembly site for multiprotein complexes. After tyrosine phosphorylation of FRS2α, the protein tyrosine phosphatase Shp2 is recruited, which allows the phosphorylation of Shp2 to promote the binding of FRS2α to growth factor receptor-binding protein 2 (GRB2) and SOS proteins to form a complex, which in turn activates the RAS-MAPK signaling pathway (Eswarakumar et al., 2005). Besides, tyrosine phosphorylation of FRS2α also mediates the recruitment of GRB2 and GAB1, which leads to the activation of the PI3K-AKT signaling pathway (Ong et al., 2001). Activated PLC-γ catalyzes the hydrolysis of phosphatidylinositol 4,5-bisphosphate (PIP2) to generate two effectors, namely inositol 1,4,5-trisphosphate (IP3) and diacylglycerol (DAG), IP3 is involved in the regulation of calcium channels in the endoplasmic reticulum. At the same time, DAG mediates the activation of protein kinase C (PKC) and other downstream targets (Kadamur and Ross, 2013).

The four known branches of the FGFR intracellular signal cascade are regulated by inhibitory molecules, including GRB2 protein, SEF (the similar expression to FGF), Spry protein, E3 ubiquitin ligase CBL, and bispecific phosphatase 6 (DUSP 6). The combination of PLC-γ and FGFR was inhibited by high concentration GRB2 (Timsah et al., 2014). Spry proteins inhibit the RAS-MAPK pathway and regulate the PI3K-AKT pathway by preventing the recruitment of GRB2-SOS complexes to FRS2α or Shp2 (Hanafusa et al., 2002; Pintus et al., 2013). SEF has been shown to regulate FGF-mediated ERK activation, and SEF specifically negatively binds activated MEK and inhibits dissociation of the MEK-ERK complex, thereby blocking nuclear transport of activated ERK (Torii et al., 2004). The extracellular domain of SEF may also directly interact with FGFR to inhibit receptor phosphorylation (Kovalenko et al., 2006). CBL inhibits FGFR signaling by forming a ternary complex with GRB2 and tyrosine-phosphorylated FRS2α, thereby promoting ubiquitination and degradation of FGFR and FRS2α (Wong et al., 2002). CBL also interacts with PI3K, leading to its ubiquitination and degradation (Dufour et al., 2008). DUSP6 inhibits MAPK signaling through dephosphorylation of ERK1/2 (Li et al., 2007).

## The Role of Fibroblast Growth Factor in the Pathogenesis of Parkinson’s Disease

**FIGURE 4:**
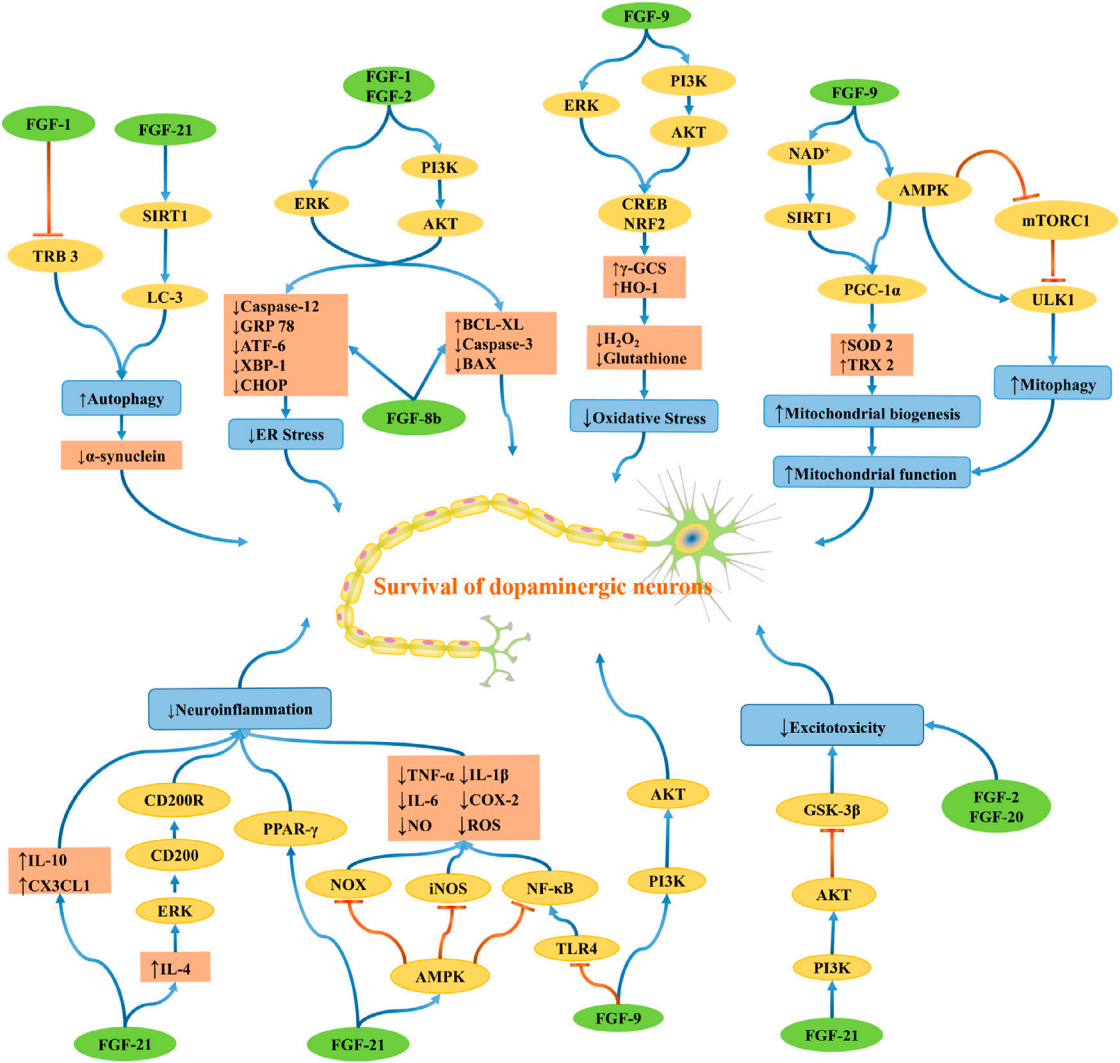
The schematic diagram depicts the molecular mechanisms by which FGF regulates oxidative stress, mitochondrial dysfunction, protein aggregation and misfolding, neuroinflammation, and excitotoxicity. Blue lines indicating positive effects, and red lines are indicating adverse effects.

PD is a complex multifactorial disease, and multiple genetic and environmental factors and their interactions are involved in the pathogenesis of PD. The possible mechanisms leading to the pathogenesis of PD include oxidative stress, mitochondrial dysfunction, protein aggregation and misfolding, neuroinflammation, excitotoxicity, apoptosis, and other cell death pathways. The development of PD is probably not caused by one mechanism but by several pathogenic mechanisms acting synergistically in a network through complex interactions, which induce dopaminergic neuron degeneration (Figure 4).

### Fibroblast Growth Factor and Protein Misfolding and Aggregation

Like any other cell, neurons are also vulnerable to misfolded proteins or other mutated proteins in the cell. The endoplasmic reticulum (ER) plays a vital role in protein folding. After the peptide is synthesized in the cytoplasm, it is transported to the endoplasmic reticulum, where chaperone proteins maintain their proper shape. However, due to mutation, over-expression, or abnormal post-translational modification, misfolding may occur. In neurodegenerative diseases such as PD, misfolded proteins are abnormally aggregated and accumulated in the endoplasmic reticulum, which is harmful to neurons. When misfolded or unfolded proteins gradually accumulate in the ER, ER stress appears and triggers the unfolded protein response (UPR). The UPR is a compensatory mechanism. Under non-stress conditions, the ER chaperone BIP protein (GRP 78) binds to and inhibits the activation of ER stress sensors (protein kinase-like ER kinase (PERK), activating transcription factor 6 (ATF6), inositol requiring protein 1 (IRE 1). When ER stress occurs, BIP preferentially binds misfolded proteins, which releases its inhibitory interaction with stress sensors, and reduces a load of unfolded protein to maintain the vitality and function of cells (Hetz, 2012). However, as misfolded proteins accumulate, they will overload the endoplasmic reticulum, leading to apoptosis.

The PERK-eukaryotic initiation factor 2 (EIF 2) -activated transcription factor 4 (ATF4) pathway is activated, which in turn regulates the expression of CCAAT enhancer-binding protein (C/EBP) homologous protein (CHOP) (Galehdar et al., 2010; Nishitoh, 2012). CHOP is the initial signal of triggering apoptosis pathway, which inhibits the promoter of the *bcl-2* gene, up-regulates apoptosis-related genes such as *caspase-12* and *caspase-3* promote cell death (Hu et al., 2018). The higher levels of phosphorylated PERK confirmed this association, and downstream eIF 2α detected in PD patients (Hoozemans and Scheper, 2012). Additionally, in human PD, the PERK immunoreactive neurons colocalize with the α-synuclein. Phosphorylated α-synuclein (Sugeno et al., 2008) and α-synuclein oligomers (Colla et al., 2012) are known inducers of ER stress, which has been well characterized in the brains of PD patients (Tsujii et al., 2015; Wang, 2016). These findings indicate that endoplasmic reticulum stress in dopaminergic neurons is positively correlated with misfolded α-synuclein.

Acidic fibroblast growth factor (aFGF) and bFGF have been shown to improve the recovery of motor function, increase the survival rate of tyrosine hydroxylase (TH) positive neurons in the substantia nigra and striatal dopamine levels by down-regulating ER stress mediators and apoptosis levels. AFGF can also reduce the accumulation of α-synuclein and reduce its neurotoxicity. The above effects may be related to the activation of PI3K/AKT and ERK1/2 signaling pathways (Wei et al., 2014; Cai et al., 2016). PI3K/Akt mediates the phosphorylation of Bax, a pro-apoptotic Bcl-2 family member, which inhibits apoptosis and promotes cell survival (Sanchez et al., 2012). Fibroblast growth factor 8b (FGF-8b) can also exert neuroprotective effects by attenuating ER stress. FGF-8b treatment decreased the mRNA levels of the ER stress markers caspase-12 and GRP 78 and the pro-apoptotic genes *caspase-3* and *Bax*, while the mRNA levels of the anti-apoptotic gene *Bcl-xl* were significantly up-regulated, confirming that FGF-8b has the effect of inhibiting apoptosis and protecting cells from ER stress (Chen et al., 2016).

Maintaining an average balance between the formation and degradation of proteins in cells is also necessary for cell survival. The ubiquitin-proteasome system (UPS) and autophagy-lysosome pathway (ALP) are the primary degradation pathways on which neurons maintain proteostasis. The former tends to eliminate soluble proteins with short half-lives, while the latter uses insoluble protein aggregates as substrates. Under normal conditions, the main degradation pathway of α-synuclein *in vivo* is the UPS. Concurrently, ALP is activated with increasing α-synuclein levels (Ebrahimi-Fakhari et al., 2011), caused by translocation of the transcription factor EB (TFEB, a central transcriptional regulator of the autophagy-lysosomal pathway) to the nucleus (Decressac et al., 2013). Mutant α-synuclein has a high affinity for lysosomal membrane receptors that mediate the autophagic pathway, preventing lysosomal uptake and inhibiting ALP from degrading them (Pan et al., 2008). The mutant α-synuclein binds to TFEB and remains in the cytoplasmic inclusion bodies, preventing it from transferring to the nucleus, thereby preventing TFEB-induced ALP activation (Decressac et al., 2013). Enhancing autophagy reduces the toxic effects of α-synuclein mutations on midbrain dopaminergic neurons (Decressac et al., 2013).

It has been found that aFGF can play its neuroprotective role in PD by inhibiting ER stress and down-regulating apoptosis-promoting protein TRB3, thus activating autophagy and reducing α-synuclein accumulation (Zhong et al., 2019). TRB3 is mainly induced by the ATF4-CHOP pathway, and it is related to apoptosis induced by endoplasmic reticulum stress (Ohoka et al., 2005). Aimé et al. found that TRB3 is overexpressed in patients with PD and cell models of PD and leads to degeneration and death of dopaminergic neurons by reducing Parkin protein expression (Aime et al., 2015). Also, inhibition of autophagy leads to ER stress, which ultimately activates the transcription factor ATF4, thereby maintaining neurons’ health and survival during stress (Kakoty et al., 2020). ATF4 is also a factor known to activate fibroblast growth factor 21 (FGF-21), which has significant neuroprotective effects (De Sousa-Coelho et al., 2012).

Experiments have demonstrated that FGF-21 ameliorates dopaminergic neuron loss and α-synuclein pathological abnormalities *in vivo* and *in vitro* models of PD, and the SIRT1-autophagy axis plays an essential role in FGF-21 induced α-synuclein clearance (Chen et al., 2020). SIRT1 is an NAD^+^-dependent deacetylase that can affect multiple targets, including LC3 and PGC-1α. Activated SIRT1 promotes autophagy degradation of α-synuclein through deacetylation of LC3 (Guo et al., 2016). Aggregation of α-synuclein can also cause severe mitochondrial damage and exacerbate oxidative stress, which leads to neurodegeneration, and enhanced autophagy can remove initially damaged mitochondria and aggregated α-synuclein and prevent oxidative stress induced by damaged mitochondria and aggregated α-synuclein (Giordano et al., 2014).

### Fibroblast Growth Factor Improves Oxidative Stress and Mitochondrial Dysfunction

Accumulating evidence suggests that mitochondria have become an attractive target for neuroprotection in patients with PD (Schapira and Patel, 2014). Mitochondria are crucial organelles, which produce ATP through oxidative phosphorylation, providing most of the energy needed for cell function. As a by-product of oxidative phosphorylation, reactive oxygen species (ROS) are produced in mitochondria. With the passage of time, ROS will damage mitochondria and weaken their function (Surmeier et al., 2017). Oxygen radicals act on mitochondrial respiratory chain complex I leads to its deficiency, and the leakage of electrons through the respiratory chain leads to the increase of ROS production, which leads to a vicious circle of intensified mitochondrial dysfunction (Franco-Iborra et al., 2016).

Experiments have also demonstrated that inhibition of mitochondrial electron transport chain complex I and oxidative stress can lead to dopaminergic cell loss and PD *in vivo* (Abou-Sleiman et al., 2006). In addition to PD, ER stress, oxidative stress, and mitochondrial damage are closely related. ER stress can lead to oxidative damage by activating the function of ER oxide protein ERO-1, which is involved in disulfide bond formation during ER protein folding to alleviate ER stress, and glutathione helps reduce them when incorrect disulfide bonds are formed, but this also leads to the reduction of glutathione (Bhandary et al., 2012). With the depletion of glutathione, ROS in mitochondria increased, causing mitochondrial damage and eventually leading to cell death (Chen et al., 2011). Mitochondrial stress also induces ER stress, which is reflected in the induction of the UPR (Bouman et al., 2011).

It has been found that FGF-9 treatment alone leads to decreased hydrogen peroxide (H_2_O_2_) levels and increased glutathione content in primary cortical neurons, which can alleviate oxidative damage by up-regulating the expression of antioxidant enzymes, such as heme oxygenase 1 (HO-1) and γ-glutamylcysteine synthase (γ-GCS), and playing a vital role in antioxidant and neuroprotective (Huang and Chuang, 2010). FGF-9 activates two parallel downstream ERK1/2 and AKT signaling pathways by combining FGFR, enhances the transcription activity of nuclear factor E-2 related factor 2 (NRF2) and cAMP response element binding protein (CREB), and up-regulates the expression of γ-GCS and HO-1, thus promoting the survival of neurons and protecting neurons from MPP^+^-induced oxidative damage *in vivo* and *in vitro* (Chuang et al., 2015).

Some genes have been identified as the single-gene causes of familial PD, and many pathogenic mutations in these genes are directly related to mitochondrial dysfunction, including α-synuclein gene (*SNCA*), *Parkin*, *DJ-1* (a gene related to early-onset autosomal recessive PD), *PINK 1* (PTEN-induced kinase 1) and *LRRK 2* (leucine-rich repetitive kinase 2) (Abou-Sleiman et al., 2006). It has been found that mutations of *SNCA* and *Parkin* genes inhibit the expression of peroxisome proliferator-activated receptor-γ coactivator-1α (PGC-1α), hinder mitochondrial biogenesis, and eventually lead to mitochondrial dysfunction and cell death (Ryan et al., 2013; Stevens et al., 2015). PGC-1α is a transcriptional coactivator, the primary regulator of cell metabolism, mitochondrial biogenesis, oxidative stress, and gene expression (O'Hagan et al., 2009). A meta-analysis of patients with PD reported the decrease of PGC-1α and its downstream genes in the disease and confirmed that PGC-1α signaling was a potential target for early intervention in PD (Zheng et al., 2010).

Studies have shown that adding fibroblast growth factor 21 (FGF-21) to cultured human dopaminergic neurons can promote the expression of nicotinamide adenine dinucleotide (NAD+) and SIRT 1 in cells and improve the level and activity of PGC-1α. After activation of PGC-1α, the levels of antioxidant enzymes such as thioredoxin 2 (TRX 2) and superoxide dismutase 2 (SOD 2) increased, and the respiratory capacity of mitochondria increased, thus improving mitochondrial efficacy and cell survival (Mäkelä et al., 2014). It has also been found that FGF-21 can rescue the MPTP-induced decrease in mitochondrial DNA copy number, which in turn stimulates the expression of electron transport chain marker genes and enhances mitochondrial function by stimulating the AMPK/PGC-1α axis (Fang et al., 2020).

AMPK is an upstream effector of PGC-1α (Wan et al., 2014) and promotes mitochondrial biogenesis by improving PGC-1α transcriptional and post-translational phosphorylation (Fernandez-Marcos and Auwerx, 2011; Scarpulla, 2011). AMPK is the principal sensor of intracellular energy stress, which can dynamically regulate the fusion and division of mitochondria according to cells’ energy state and help dilute and isolate damaged mitochondria. When there is a slight energy deficiency, mitochondria fuse to form tubular networks, maximizing energy production. However, in more severe cell stress, AMPK induces mitosis and mitosis (Zhang and Lin, 2016). It has been pointed out that AMPK activates autophagosome formation by phosphorylating ULK 1 and alleviates its inhibition of ULK 1 by inhibiting MTORC 1, promotes mitophagy, removes damaged or dysfunctional mitochondria, and protects dopaminergic neurons (Mihaylova and Shaw, 2011).

### The Role of Fibroblast Growth Factor in Neuroinflammation

The role of FGF in neuroinflammation is one of the pathological characteristics of PD (Hassanzadeh and Rahimmi, 2018). Activated microglia have been found in the substantia nigra and putamen of patients with PD (Iannaccone et al., 2013). In response to infection or injury, microglia transform into the reactive inflammatory phenotype, also known as classical activation or M1 phenotype, which is characterized by increased proliferation, morphological changes, and release of inflammatory molecules such as cytokines, chemokines, and reactive oxygen species (Kettenmann et al., 2011).

Although the M1 phenotype is designed to protect and repair the central nervous system, it can also be cytotoxic and detrimental to the neural microenvironment, causing neurodegenerative diseases if excessive and prolonged neuroinflammation is produced (Czeh et al., 2011; Cherry JD et al., 2014). For example, microglial activation leads to the activation of enzymes associated with inflammation, such as inducible nitric oxide lyase and cyclooxygenase, and the release of inflammatory cytokines, such as chemokine (CXCL 12), tumor necrosis factor-α (TNF-α), interferon-γ (IFN-γ), interleukin-6 (IL-6), and interleukin-1β (IL-1β), which leads to neural network dysfunction and promotes inflammatory responses (Rocha et al., 2015). Activation of microglia in PD is regulated by the CD200-CD200R signaling pathway (Wang et al., 2007).

Additionally, it has been suggested that different gene mutations, such as *SNCA*, *LRRK 2*, or *DJ-1*, stimulate inflammation by activating microglia and astrocytes, thus aggravating the loss of dopaminergic neurons and chronic neurodegeneration in patients with PD (Gillardon et al., 2012; Moehle et al., 2012; Harms et al., 2013; Nash et al., 2017).

FGF-2 reduced the levels of pro-inflammatory cytokines such as interleukin-1β (IL-1β), interleukin-6, and tumor necrosis factor-α (TNF-α), increased the level of anti-inflammatory cytokine IL-10 and reversed the decrease of the expression of chemokine CX3CL1, which is mainly expressed by neurons and maintained monitoring of microglia (Tang et al., 2018). A previous study also found that FGF-2 can regulate microglia activation and decrease inflammatory mediators’ expression in a CD200-dependent manner (Cox et al., 2013). Activated FGF-2 signaling regulates IL-4 production by glial cells, and IL-4 induces ERK signaling to increase CD200 expression, thereby enhancing the interaction between CD200 and CD200R and hindering microglial activation (Downer et al., 2010).

Multiple mechanisms can be induced upon microglial activation, including the NF-κB signaling pathway (He et al., 2017). NF-κB is a crucial transcription factor in the progression of inflammation, and its activation is accompanied by the release of a series of inflammatory cytokines and chemokines, such as TNF-α, IL-1β, IL-6, and Cox-2 (Zusso et al., 2019; Li et al., 2020). Experiments show that fibroblast growth factor 10 (FGF-10) can activate the PI3K/Akt survival signaling pathway and inhibit the activation and proliferation of microglia/macrophages by inhibiting the TLR4/NF-κB signaling, thereby inhibiting the production of pro-inflammatory factors (TNF-α and IL-6) and exerting neuroprotection (Li et al., 2016; Chen et al., 2017).

PGC-1α is a potential new target for treating patients with PD, and its activity is mainly regulated by PPAR-γ, AMPK, and sirtuin 1 (SIRT 1) (De Virgilio et al., 2016). Therefore, pharmacological activators of these proteins have the potential to exert their effects by activating PGC-1α. Wang et al. found that FGF-21 binds to fibroblast growth factor receptor 1 (FGFR1) and inhibits the inflammatory response by inhibiting NF-κB and up-regulating the expression of peroxisome proliferator-activated receptor-γ (PPAR-γ) (Wang J.et al., 2020). FGF-21-induced AMPK activation may also be one of the mechanisms that inhibit neuroinflammation, with AMPK inhibiting NOX-mediated ROS production, iNOS mediated NO production, and NF-κB-mediated production of pro-inflammatory cytokines such as IL-1 and TNF-α (Fang et al., 2020).

### Fibroblast Growth Factor and Excitotoxicity

Excitotoxicity has been considered as the pathogenesis of many neurodegenerative diseases, including PD. Glutamic acid is a cardinal neurotransmitter in the central nervous system of mammals, and it is a significant participant in the processes of excitotoxicity. Previous studies have shown that glutamate excitotoxicity may lead to degeneration of dopaminergic neurons and accompanying motor dysfunction in PD (Meredith et al., 2009). Glutamate receptors are abundantly found in the SN’s dopaminergic neurons and are innervated by glutamate from the thalamus and cortex. Under pathological conditions, the extracellular glutamate concentration is elevated when the presynaptic membrane releases excess glutamate, or the glutamate reuptake function is impaired. Activated microglia and reactive astrocytes release large amounts of glutamate (Iovino et al., 2020).

Extracellular excess glutamate leads to overactivation of Ca^2+^ permeable N-methyl-D-aspartate receptors (NMDARs), followed by Ca^2+^ overload and excitotoxicity (Lewerenz and Maher, 2015). Ca^2+^ influx increases the activity of nitric oxide lyase (NOS), an enzyme by which NO can react with superoxide radicals to generate ONOO-, which causes severe oxidative destruction of cellular contents and impairs cellular energy production, ultimately inducing cell death (acute necrosis and/or delayed apoptosis) (Wang D. et al., 2020). The prominent role of the glutamine/glutamate-γ-aminobutyric acid cycle (GGC) is to regulate synaptic glutamate levels, which prevent excitotoxicity and maintain normal central nervous system function. GGC disorders lead to alterations in glutamatergic and gamma-aminobutyric acidergic neurotransmitter pathways associated with PD (Gao et al., 2013). It has been established that elevated Gln, Glu, and GABA were significantly reduced to normal levels in PD rats after FGF-2 treatment, suggesting that FGF-2 can help maintain homeostasis GGC, thereby preventing and treating PD (Zheng et al., 2016). FGF-21 protects primary central nervous system neurons from glutamate excitotoxin-induced apoptosis, and its mediated neuroprotection is achieved by activating the cytoprotective factor AKT-1 as inhibiting the activity of the cytotoxic factor GSK-3β (Leng et al., 2015).

Another method to improve excitotoxicity is to solve downstream processes, including intracellular calcium-related signaling systems. Based on clinical data obtained from postmortem brains of PD patients, dopaminergic neurons in substantia nigra expressing calcium-binding protein calbindin (CB) selectively inhibit cell death damage (Inoue et al., 2019). CB is ubiquitously expressed in many brain regions and is involved in regulating intracellular Ca^2+^ levels (Blesa and Vila, 2019). In PD, calcium-binding protein-negative dopaminergic neurons are preferentially lost, while FGF-20 rescues calcium-binding protein-negative midbrain dopaminergic neurons from cytosol dopamine toxicity induced by 6-OHDA and stress and promotes dopamine release of calcium-binding protein-negative dopaminergic neurons by activating FGFR1 and then activating its downstream cascade (Murase and McKay, 2006).

## Fibroblast Growth Factor Plays an Essential Role in Protecting and Repairing Dopaminergic Neurons

Dopaminergic neuron apoptosis is a characteristic of PD and preventing dopaminergic neuron apoptosis is considered as an effective strategy to treat Parkinson’s syndrome (Parmar, 2018). FGF-2 regulates dopaminergic neurons’ development and the nigrostriatal pathway *in vivo*, which is the main pathway affecting human beings in PD (Baron et al., 2012). Studies have shown that reactive astrocyte FGF-2 levels are increased during 6-OHDA induced degeneration of nigrostriatal dopaminergic neurons in rats, suggesting that increased astrocyte FGF-2 synthesis may be related to neuronal repair processes (Silva et al., 2009).

FGF-2 further demonstrated the importance of FGF-2 in the viability of dopaminergic neurons in the substantia nigra of a mouse model of PD by improving their survival and protecting them from 6-OHDA-induced cell death (Grothe and Timmer, 2007). FGF-2-deleted mice showed a significant decrease in dopaminergic neuron survival after nigral injury with 6-hydroxydopamine, and the number of dopaminergic neurons was regulated by FGFR3 (Timmer et al., 2007). The activity of the FGF-2-PI3K/AKT signaling axis is required for neural survival and plasticity. When the signaling pathway is activated, it up-regulates the anti-apoptotic protein Bcl-2. It inhibits the activation of the pro-apoptotic enzyme caspase-3, thereby inhibiting apoptosis and promoting cell survival, protecting the cell body’s integrity and neurite branching from MPP^+^-induced toxicity (Yu et al., 2019). FGF-2 is not effective in all research. In the study by Jaumotte et al. FGF-2 cannot protect DA neurons from MPP^+^, but the combined action of various neurotrophic factors has protective effects, which may be related to different PD models (Jaumotte et al., 2016).

FGF-8 is also a promising candidate for the treatment of neurodegenerative diseases, where it has broad activity in neural tissue and is vital in promoting dopaminergic neuron development and function (Chen et al., 2016). FGF-8 can induce dopaminergic neuronal differentiation and promote dopaminergic axons’ growth in the midbrain by increasing Semaphorin 3F (Yamauchi et al., 2009; Lim et al., 2015). Two major FGF-8 isoforms are expressed in the midbrain (FGF-8a and FGF-8b), whereas FGF-8 b promotes midbrain development, and FGF-8b is structurally similar to FGF-18 and has similar receptor-binding characteristics (Liu et al., 2003). FGF-18 has been proved to protect dopaminergic neurons in substantia nigra and may be used as a neuroprotective agent in PD. Intrastriatal infusion of FGF-18 prevents dopaminergic neuron loss in the substantia nigra and significantly improves motor dysfunction in a 6-OHDA-induced rat model of PD. In addition, results from *in vitro* studies suggest that the AKT/GSK-3β signaling pathway is involved in the neuroprotective effects of FGF-18 against 6-OHDA-induced neurotoxicity (Guo et al., 2017).

It has also been shown that endogenous FGF-9 is a survival factor for dopaminergic neurons and that FGF-9 treatment of cultured substantia nigra and midbrain cells prevents MPP^+^-induced dopaminergic neuron death (Huang et al., 2009).

FGF-20, a member of the FGF-9 subfamily, is preferentially expressed in the adult brain and has the highest expression levels in the cerebellum and substantia nigra. FGF-20 protects dopaminergic neurons from a series of toxic injuries *in vitro* by activating fibroblast growth factor receptor 1 (FGFR1), which significantly improves the survival rate of cultured dopaminergic neurons (Sleeman et al., 2012). The binding of FGF-20 to FGFR1c induces phosphorylation of specific cytoplasmic tyrosine residues, thereby activating the mitogen-activated protein kinase (MAPK) pathway is essential for the survival of dopaminergic neurons (Itoh and Ohta, 2013). Moreover, infusion of FGF-20 *in vivo* protects dopaminergic neurons from 6-OHDA-induced damage. It prevents loss of dopaminergic neurons in the substantia nigra and subsequent dyskinesia in PD rats (Sleeman et al., 2012), endogenous FGF-20 is produced by astrocytes and diffuses in a paracrine manner to neighboring dopaminergic cells within the substantia nigra to provide protection (Boshoff et al., 2018).

## The Role of Fibroblast Growth Factor in the Neural Differentiation of Stem Cells Into Dopaminergic Neurons

Embryonic stem cells (ESCs) are pluripotent cells, which originate from cell population differentiation in the blastocyst stage. These cells have many characteristics of the cell origin needed for cell replacement therapy, including proliferation and differentiation ability. Direct differentiation of ESCs into dopaminergic neurons has been realized (Ganat et al., 2012), which may provide a source of cell transplantation therapy for the treatment of PD (Kriks et al., 2011). The main challenge of improving embryonic stem cells’ therapeutic effect is to promote the proper differentiation and long-term survival in brain regions, which are susceptible to neurodegeneration in PD.

As a physiologically relevant neurotrophic factor, FGF-2 plays an important role in embryonic development and neural differentiation of embryonic stem cells and is one of the key factors determining the differentiation of dopaminergic neurons in human embryonic stem cells (hESCs) (Cho and Kim, 2008; Lahti et al., 2012). It has also been shown that FGFR synergistically regulates the self-renewal of nerve progenitor cells and the differentiation of dopaminergic neurons during midbrain development (Saarimaki-Vire et al., 2007). In the adult brain, FGF-2 is mainly synthesized and secreted by astrocytes (Zhang et al., 2009).

Experiments show that the *in situ* release of astrocyte-specific FGF-2 is promoted by specific activation of endogenous astrocytes in the substantia nigra, which significantly enhances dopaminergic neuron differentiation and brain function repair of transplanted hESCs in PD rat model (Yang et al., 2014). Sonic hedgehog (Shh) and FGF-8 have been used specifically to differentiate ESCs into tyrosine hydroxylase-positive neurons *in vitro*. When both Shh and FGF-8 exist, developing cells *in vivo* differentiate into a dopaminergic neuron phenotype when they encounter cross signals along the anterior-posterior (FGF-8) and dorsal-abdominal (Shh) axes (Nandy et al., 2014). FGF-20 synergizes with FGF-2 to increase the number of dopaminergic neurons in primate ESC-derived neurons composed of neural progenitor cells, and transplantation of the resulting dopaminergic neurons into a primate model of MPTP-induced PD can act as dopaminergic neurons and reduce the neurological symptoms caused by MPTP (Itoh and Ohta, 2013).

Mesenchymal stem cells are pluripotent stem cells. Compared with ESCs, mesenchymal stem cells have the characteristics of easy harvesting, no ethical issues, and the potential of autologous transplantation. An *in vitro* study has shown that co-culture of ventral midbrain cells and rat bone marrow mesenchymal stem cells (BMSCs) can enhance tyrosine hydroxylase expression and dopamine synthesis (Jin et al., 2008). Several *in vivo* studies have shown that the implantation of intrastriatal BMSCs promotes functional recovery in a rat model of Hemi-PD (Deierborg et al., 2008; Delcroix et al., 2011). Transplantation of BMSCs can alleviate the dyskinesia of animal models of PD, but the effect is limited, and only a few transplanted cells can survive in the brain of the host after transplantation.

FGF-2 alone is an effective inducer of differentiation of bone marrow mesenchymal stem cells into functional dopaminergic neurons (Nandy et al., 2014). FGF-2 can promote the neural differentiation of human bone marrow mesenchymal stem cells (hBM-MSCs) *in vitro* and *in vivo*, and FGF-2 supplementation can enhance the cell viability and proliferation ability of hBM-MSCs and improve the therapeutic effect (Xiong et al., 2013). Furthermore, human umbilical cord mesenchymal stem cells (hUC-MSCs) derived from the human umbilical cord also have great potential in the treatment of PD. Introduction of FGF-20 gene into hUC-MSCs and transplantation into a mouse model of PD significantly improved mouse behavior, accompanied by an increase in tyrosine hydroxylase-positive cells and dopaminergic neurons, which may be related to MSC-FGF-20 promoting the degradation of the transcription factor NF-κB in the nigrostriatal dopaminergic system (Jinfeng et al., 2016).

## Neurotrophic Factors as Therapy Strategies for Parkinson’s disease

Neurotrophic factors, such as GDNF and BDNF, have been proved to have considerable therapeutic potential in neuroprotection and nerve recovery in PD, because they can promote the growth and survival of dopaminergic neurons and enhance their functional activity (Allen et al., 2013; Kowianski et al., 2018). In recent years, the potential of neurotrophic factors to protect nigrostriatal neurons in PD has been extensively explored. Many neurotrophic factors have entered clinical trials, but they have failed to provide significant clinical improvement for patients with PD. Some preclinical and clinical data show that increasing GDNF concentration does not always lead to significant long-term improvement. Thus, there has been uncertainty about the value of neurotrophic factors in the future treatment of PD (Lindholm et al., 2016; Ferreira et al., 2018). The macromolecular size of neurotrophins poses a great challenge for drugs to cross the blood-brain barrier (BBB) and specifically target diseased brain regions (Nagahara and Tuszynski, 2011; Barker et al., 2020). It is difficult for macromolecules to pass through the BBB, so they must be administered by intraventricular or intrathecal infusion. Extensive central administration may lead to serious side effects, such as epilepsy, sensory disturbance, and Schwann cells migration/proliferation to subpial space (Nagahara and Tuszynski, 2011). Furthermore, the treatment of many nervous system diseases requires local and continuous delivery of growth factors (Allen et al., 2013). How to achieve a balance between adequate infusion (neurotrophic factors are effectively distributed to target sites without damaging tissues) and excessive infusion (which may lead to side effects) is a significant obstacle in the clinical translation of PD growth factor therapy (Whone et al., 2019).

FGF-21 is an endocrine hormone, which has various effects on metabolism regulation. It has been shown that FGF-21 is expressed in different regions of the brain, especially in midbrain regions containing dopaminergic neurons (Mäkelä et al., 2014). Potential receptors of FGF-21 are widely distributed in the central nervous system (Fon Tacer et al., 2010), and it has been reported that FGF-21 plays different roles in the central nervous system (Bookout et al., 2013; Chen et al., 2019). More interestingly, FGF-21 in the periphery can cross the BBB by simple diffusion, reach the brain directly to exert neuroprotective effects (Hsuchou et al., 2007), and be detected in the cerebrospinal fluid of humans and rodents (Liang et al., 2014). These evidence show that FGF-21 can act directly on the central nervous system and has great potential in treating PD. In addition, FGF-2 has been studied to improve its pharmacological activity, covalently linking polyethylene glycol (PEG) polymer. Compared with native FGF-2, polyethylene glycol-modified FGF-2 achieves better BBB permeability and *in vivo* stability, thereby improving its transport (Zhu et al.,). Nasal administration is also an effective method for the treatment of central nervous system diseases. Nasal administration of liposome-loaded bFGF significantly reduced behavioral impairment and rescued the 6-OHDA-induced loss of TH-positive neurons in PD model rats (Yang et al., 2016). Notably, although the ease of administration is a clear advantage of intranasal administration, the potential off-target effects of this route of administration may limit its clinical translation (Bender et al., 2015). Recently, focused ultrasound (FUS), which can reversibly open the BBB in a site-specific manner, has been experimentally established as a non-invasive and localized brain drug delivery technology (Aryal et al., 2014). Niu et al. protected a rat model of 6-OHDA-induced PD by focusing ultrasound-guided systemic administration of recombinant human FGF-20 proteolipids by fusing small ubiquitin-associated modifier (SUMO) to rhFGF-20 to enhance the efficiency of its soluble expression (Niu et al., 2018). Another method is to use small molecule agonists to target related receptors and specifically activate neurotrophin signals. The effects of existing drugs on endogenous FGF 20 production in substantia nigra and striatum were studied. It was finally determined that salbutamol and trifluorofloxacin could be used to increase the FGF-20 level to resist the progression of Parkinson's disease (Fletcher et al., 2019).

## Conclusions and Future Research Directions

The pathogenesis of PD is not the result of dysfunction of a specific pathway, but a combination of a series of interrelated pathogenic event and dealing with these pathogenic mechanisms alone may not be sufficient to prevent neurodegeneration. Another method is to increase the survival rate of vulnerable neurons by increasing neurotrophic factors. Many studies have demonstrated the critical role of FGF and its receptors in neuroprotection and neurogenesis in PD. In PD models, FGF provides adequate protection against the loss of dopaminergic neurons, promotes differentiation of cultured cells into dopaminergic neurons in PD animal model, and alleviates neurological symptoms. These findings indicate that FGF plays an important role in the differentiation and survival of dopaminergic neurons and the etiology and treatment of PD. It is worth noting that most of the beneficial effects of FGF observed at present are obtained from drug-induced PD models. Thus, whether FGF plays a role in actual diseases is still unclear. Further clinical studies are needed to evaluate the safety and effectiveness of FGF in the treatment of PD.

The BBB poses another challenge to the use of macromolecules (e.g., growth factors) to treat neurodegenerative diseases. The BBB is a double-edged sword that effectively protects the brain from foreign bodies, but it also limits the use of many therapeutic agents aimed at treating neurological disorders. At present, gene therapy *in vivo* is still the only way to achieve clinical trials by directly injecting growth factor protein into the affected brain regions, and they are still the most promising solutions. However, the latest advances in gene therapy and biomaterial-assisted protein and gene delivery make the alternative growth factor delivery systems closer to clinical trials. Gene therapy plays a therapeutic role by genetically engineering cells *in vitro* to produce neurotrophic factors and then transplanting them back into patients (Gowing et al., 2017). The main advantages of gene therapy is that genetic engineering is carried out *in vitro*, gene vectors are not directly injected into the patient's brain, and genetically engineered cells can be rigorously evaluated before clinical practice. In addition, thesafety of PD gene therapy *in vivo* has been proved in many clinical trials (Hitti et al., 2019). As a new therapeutic method, the selection of gene vectors, the nature of cell donors, cell types, and drug delivery routes are the key factors to be considered in the delivery of growth factors *in vitro* for PD (Gowing et al., 2017). Biomaterial-assisted growth factor delivery also has excellent potential in PD therapy. Liposomes and other biomaterial particles protect proteins and genes from destruction by *in vitro* and *in vivo* factors and have been shown to improve brain penetration of growth factor proteins and gene therapy after FUS therapy in animal models of PD (Mead et al., 2017; Price et al., 2019; Umlauf and Shusta, 2019). At present, the first clinical trial (NCT 03608553) of FUS opening BBB for PD is underway, and the time will determine whether this method is safe and effective. In addition, new compounds that specifically activate FGF signaling in dopaminergic neurons or specifically target these neurons may prove helpful in PD treatment. The identification of dopaminergic-specific FGF interactors may be helpful for the screening of such compounds. The role of FGF in PD and the development of therapeutic interventions deserve further studies. More research is needed to explore the appropriate therapeutic window, dosage, and combination with other therapeutic agents or biomaterials, which will help promote the clinical application of FGF.
